# Using intervention mapping to deconstruct cognitive work hardening: a return-to-work intervention for people with depression

**DOI:** 10.1186/s12913-014-0530-4

**Published:** 2014-12-12

**Authors:** Adeena Wisenthal, Terry Krupa

**Affiliations:** Queen’s University, School of Rehabilitation Therapy, Kingston, ON Canada

**Keywords:** Intervention mapping, Cognitive work hardening, Return-to-work, Depression, Program planning

## Abstract

**Background:**

Mental health related work disability leaves are increasing at alarming rates with depression emerging as the most common mental disorder in the workforce. Treatments are available to alleviate depressive symptoms and associated functional impacts; however, they are not specifically aimed at preparing people to return to work. Cognitive work hardening (CWH) is a novel intervention that addresses this gap in the health care system. This paper presents a theoretical analysis of the components and underlying mechanisms of CWH using Intervention Mapping (IM) as a tool to deconstruct its elements.

**Methods:**

The cognitive sequelae of depression and their relevance to return-to-work (RTW) are examined together with interpersonal skills and other work-related competencies that affect work ability. IM, a tool typically used to create programs, is used to deconstruct an existing program, namely CWH, into its component parts and link them to theories and models in the literature.

**Results:**

CWH has been deconstructed into intervention elements which are linked to program performance objectives through underlying theoretical models. In this way, linkages are made between tools and materials of the intervention and the overall program objective of ‘successful RTW for people with depression’. An empirical study of the efficacy of CWH is currently underway which should provide added insight and understanding into this intervention.

**Conclusions:**

The application of IM to CWH illustrates the theoretical underpinnings of the treatment intervention and assists with better understanding the linkage between intervention elements and intervention objective. Applying IM to deconstruct an existing program (rather than create a program) presents an alternate application of the IM tool which can have implications for other programs in terms of enhancing understanding, grounding in theoretical foundations, communicating program design, and establishing a basis for program evaluation and improvement.

## Background

Cognitive work hardening (CWH) is a treatment intervention for preparing people off work due to depression to return to work following a disability leave [1,2]. It is based on the same principles as ‘classical work hardening’ (discussed in the following section on Principles of work hardening) but applies the concepts to the mental health domain [1]. CWH fills a gap in the health care system where there has been targeted intervention to prepare for return to work (RTW) following an injury (e.g., physical work hardening) but there has been no comparable intervention in the mental health domain.

The need for a specific intervention to address the RTW needs of people with depression is supported by evidence related to the prevalence of mental illness, and depression in particular, and its impact on work productivity. In Canada, mental health disorders account for 25% of all diseases with 13% attributable to depression alone [3]. The World Health Organization predicts that by 2020, depression will be the leading cause of disability worldwide, second only to heart disease [4]. Lost productivity per year due to mental health problems in Canada has risen from $14 billion (1999) to $33 billion (2004) with mental illness related disability claims accounting for one third of workplace claims – approximately 70% of workplace costs [3-6]. Dewa et al. [3] studied the rising costs associated with poor mental health among workers internationally. They found similar trends in costs due to lost productivity and absenteeism resulting from mental health problems among many countries such as Sweden (more than 2/3 of costs), The Netherlands (€1.44 billion annually), England (30% of absences due to stress), and the United States (average depression-related absenteeism productivity loss is equivalent to $8.3 billion).

Depression is one of the most common mental disorders in the workforce [7,8]. It impacts an employee’s health, functioning, life satisfaction, and overall self-esteem. It typically results in decreased energy, fatigue, poor sleep, diminished/loss of appetite, feelings of worthlessness, hopelessness, and apathy [9,10]. Many cognitive deficits are associated with depression including impairments in concentration, memory, attention, and decision making. These problems can interfere with the ability to meet the cognitive, emotional and behavioural demands of a job [11-13]. In addition, these forms of work disability can contribute to the stigma of mental illness that is prevalent in workplaces, fuelling the assumption that competence to work is compromised [14,15].

A person no longer able to work due to depression may go on a medical disability leave. During this time, treatment may be available in the form of psychotropic medications, psychotherapy, and psychological support. These treatments can be effective in helping to alleviate depression and its functional impacts. They are not, however, specifically aimed at preparing the person to return to work.

CWH addresses this intervention gap by targeting a broad range of functional issues that people with depression face in their workplaces which are critical for resuming job duties and achieving RTW success. These include the cognitive skills required to assume job duties and meet work demands, the coping skills required to manage work-related stress and deal with interpersonal issues, and the overall stamina and functional ability to adopt a work routine and adhere to a work schedule [1,2].

The purpose of this paper is to formalize a structured framework for CWH by integrating theory and practice using the structured process of Intervention Mapping (IM). The IM process provides a systematic framework for planning, designing, and implementing health promotion programs [16,17]. This paper shows how IM can also be used to deconstruct an existing intervention for the purpose of analysis, communication, and improvement. The analysis in this paper has resulted in a structured and detailed program theory for the CWH intervention consistent with the goals of program theory evaluation [18].

There are several approaches for program evaluation [18-20]. IM was chosen for the current program analysis because its inherent structured step-wise approach is ideal for making linkages between program elements and expected program outcomes. Furthermore, IM has been used by other researchers to tailor programs for targeted groups (e.g., mental disorders) as well as targeted domains (e.g., occupational health) [21,22] both directly relating to the target population served by the CWH intervention. Empirical research is currently being conducted to study the efficacy of CWH which should complement the current analysis.

### Principles of work hardening

CWH is grounded in the principles of classical work hardening which was developed in the 1970’s to address the needs of injured workers [23-27]. While classical work hardening is applicable to a wide range of disabilities, in practice it has almost exclusively been applied to people with physical injuries. For the purpose of this paper, the term ‘physical work hardening’ (PWH) refers to the application of work hardening to this population.

Both physical and cognitive forms of work hardening are rooted in common underlying principles while the differences are based on the functional and work-related needs of the population they specifically address. Both forms of work hardening use graded work activities to simulate a person’s actual work tasks and demands. They both aim to improve a client’s work performance skills to enable the safe and productive return to the workforce [1,2,23-27].

The goals of graded work activities in PWH encompass the neuromuscular aspects of an injury; in CWH, graded work activities focus on the cognitive aspects of the client’s condition. RTW outcomes are enhanced in PWH by increasing physical endurance, pain management, injury prevention, pacing and application of ergonomic principles [26-28] while they are enhanced in CWH by increasing mental stamina, mental fatigue management, coping skills, as well as pacing, and application of ergonomic principles [1,2].

Table 1 illustrates how the elements of both forms of work hardening compare in terms of physical versus cognitive components. For example, managing one’s pain is often a critical RTW success factor for people with musculoskeletal problems [28] whereas (mental) fatigue management is critical among people returning to work following depression [29,30]. Both affect one’s ability to increase work tolerance and therefore RTW readiness. In addition, an education component is common for both these work hardening populations. PWH clients learn about injury prevention techniques and CWH clients benefit from coping skills training. Both groups benefit from education on pacing and ergonomic principles as these have been found to impact employee functioning, productivity, and mental health [31-33].

**Table 1:** **Overall comparison of physical work hardening and cognitive work hardening**

	**Physical work hardening**	**Cognitive work hardening**
**Identified needs**	Prepare injured workers to RTW	Prepare employees with depression to RTW
**Program outcome**	Facilitate a safe (and preferably early) RTW	Facilitate a safe (and preferably early) RTW
**Main elements**	Individualized	Individualized
	Physical conditioning	Cognitive skills conditioning
	Pain management	Fatigue management
	Job specific work simulations	Job specific work simulations
	Education – injury prevention, pacing, ergonomics	Education – coping skills/strategies, pacing, ergonomics

### Cognitive work hardening

CWH is a multi-element intervention, typically offered by an occupational therapist (OT) in a simulated work setting preferably in the community away from a clinic environment [1,2]. Consistent with principles of occupational therapy practice, the intervention is grounded in the analysis of the client’s job and the work environment in which the client works. Key occupational performance issues are identified by the client in an intake interview (and supported by medical documentation and other file data) and typically include fatigue, cognitive impairments (e.g., poor concentration, reduced memory, difficulty multitasking), interpersonal issues (e.g., effective communication, conflict management), and reduced coping skills (e.g., time management, organizational skills, goal setting). These performance issues are matched to work demands obtained from a job description and form the basis of the CWH intervention rendering the strategies selected individualized, highly relevant and enabling of a collaborative relationship with the client served.

In addition to the building of key cognitive and coping skills, interpersonal and organizational competencies to deal with work-related situations are typically addressed. Educational components support the development of effective coping skills, communications strategies, and approaches to facilitate the handling of workplace issues. Interventions that have an educational component contribute to one’s sense of well-being, sense of control and empowerment which can contribute to success at work [34,35].

A structured work schedule is a critical component of CWH. It provides the client a routine of meaningful and work-related activities which is typically lacking when off work on disability. Structure to the day can assist with improving feelings of self-esteem, enhancing motivation and offsetting general feelings of hopelessness that are often reported by people who experience mental illness [36]. The progressive work schedule that is inherent in CWH, together with the gradual increase in cognitive skill development, enables the client to build stamina and the levels of energy required to match work demands. Since fatigue and reduced energy are commonly associated with depression and have been found to have a negative effect on work performance [29,37], stamina and energy are addressed in CWH with close attention to the balance of work-life demands and the importance of maintaining meaningful activity participation outside of the realm of the workplace.

Through the CWH process clients become aware of their functioning and gain insight into their strengths and limitations, which can have implications for RTW. More specifically, coping skills are developed, stamina is enhanced, a routine is established, and other work-related skills are gained thereby preparing the client to meet work demands while experiencing success within the CWH simulated work environment. These gains provide the client with self-confidence to return to work. Feeling that one has the competence and ability to perform one’s job can contribute to self-efficacy, which has been found to be an essential factor in the work rehabilitation process impacting RTW outcomes [38,39]. Indeed, acquiring self-efficacy can improve self-esteem and reduce self-stigma which is often a barrier to social activity and employability [40].

## Methods

IM has been used to create new programs to address problems in health care either through original design [21,41-43] or by adapting an existing intervention to meet the needs of a different population [22,44,45]. In particular, some researchers have used the IM approach to develop workplace interventions for employees on leave due to mental disorders based on existing programs for workers with musculoskeletal conditions [22,45]. Given that CWH emerged from the more established PWH, IM is used in this paper to examine overall program and performance objectives for both forms of work hardening. The IM protocol is then used to deconstruct the CWH intervention for a more detailed analysis of its objectives, tools and materials, and linkages to the underlying theories and models providing increased insight into the application of work hardening to the mental health population.

**Figure 1:**
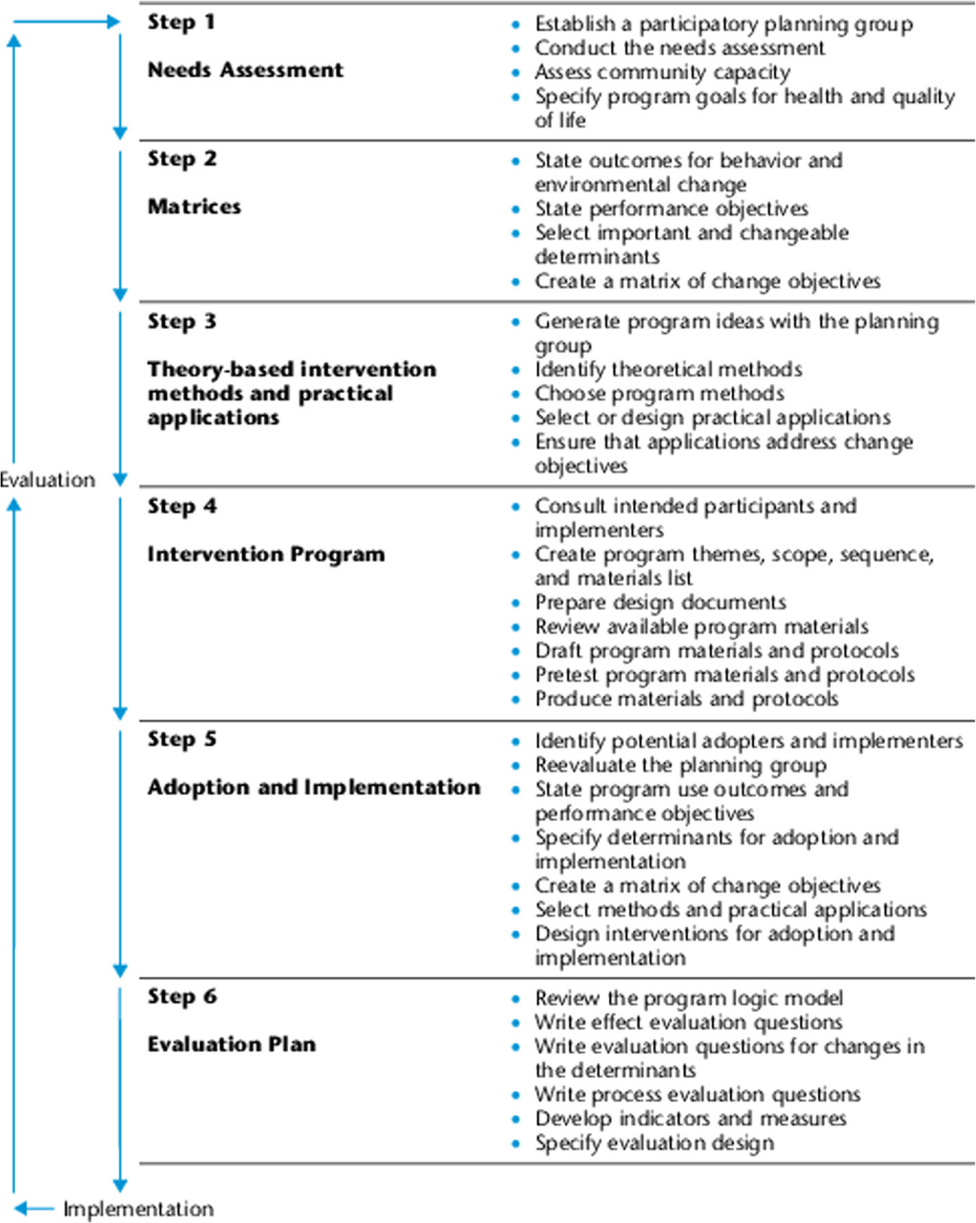
**Intervention mapping process [** 17 **].**

IM is considered a six step process with each step consisting of several tasks which, once completed, set the stage for the next step (Figure 1). The process is an iterative one, moving back and forth between tasks and steps as information and perspectives emerge from various activities. The process is cumulative with each step being based on the previous step in an effort to be comprehensive in the planning and maximize the potential effectiveness of the intervention/program [17,46].

IM Step 1 is implicit in this analysis and yields the overall program objective of ‘*successful return-to-work’* for both PWH and CWH applied to their respective populations. Step 2, Tasks 1 and 2 are used to examine the similarities and differences of these two interventions through variations in each of their overall program objectives, behavioural and environmental outcomes, and performance objectives. Step 2, Tasks 3 and 4 focus on CWH and are used to select changeable behaviour determinants of performance objectives for the behavioural and environmental outcomes with a matrix of change objectives being created that links the determinants to performance objectives. Step 3 is then used to select theoretical methods that match to the behaviour determinants and are then linked to practical strategies largely drawn from direct experience with the CWH intervention. Step 4 and Step 5 are not applicable in the current analysis because they relate to program creation and implementation whereas CWH is an existing treatment intervention. Step 6 is discussed in terms of its value in monitoring the CWH intervention under discussion, fine tuning details to enhance outcome, and contributing to research and clinical knowledge to advance health outcomes.

An ethics statement is not included in this paper as there was no study conducted and there was no research conducted on human subjects.

## Results

### IM Step 1: Needs assessment

A Needs Assessment review is not within the scope of this analysis because CWH is an existing program and its creation is not being addressed in this paper. Nevertheless, many of the elements comprising this step (e.g., discussion with stakeholders, examination of client needs) were initially used to adapt PWH to the client population served by CWH [1].

The output of this step is the overall program objective which is then used to drive the remaining IM steps. In PWH, the overall program objective is ‘*successful RTW for people with a physical injury’*. In CWH, the program objective is ‘*successful RTW for people with depression’.*

### IM Step 2: Matrices of change objectives

#### Tasks 1 and 2: Behavioural/environmental outcomes and Performance objectives

The first task in IM Step 2 is to state the behavioural and environmental outcomes that need to be achieved in order to reach the overall program objective. The next task is to specify what change is necessary in the behavioural and environmental outcomes by stating performance objectives. Performance objectives refer to the effects of the intervention in terms of behaviour that should be learned or changed (behavioural outcome) or aspects of the environment that need to be changed (environmental outcome) [17,47]. In both forms of work hardening, the behavioural outcome is ‘*Client is ready to RTW’* while the environmental outcome is *‘RTW plan is in place’*. Tables 2 and 3 present the performance objectives for PWH and CWH, respectively.

**Table 2:** **Performance objectives for behavioural and environmental outcomes: PWH**

**Program objective: Successful return-to-work for people with physical injury**	
**Behavioural outcome**	**Performance objectives**
**Client is ready to RTW**	1. Client identifies RTW barriers and accepts proposed treatment plan
	2. Client has the work tolerance to meet minimallyrequired work hours
	3. Client has physical tolerance to meet job demands
	4. Client has pain management strategies
	5. Client is aware of injury prevention strategies
	6. Client has confidence in ability to return to work
**Environmental outcome**	**Performance objectives**
**RTW plan is in place**	1. GRTW schedule is designed and agreed upon by stakeholders
	2. Workplace accommodations are provided
	3. Client’s strengths and limitations are assessed for job match

GRTW = gradual return-to-work.

**Table 3:** **Performance objectives for behavioural and environmental outcomes: CWH**

**Program objective: Successful return-to-work for people with depression**	
**Behavioural outcome**	**Performance objectives**
**Client is ready to RTW**	1. Client identifies RTW barriers, concurs with and commits to treatment plan
	2. Client has the work tolerance to meet minimally required work hours
	3. Client has cognitive skills to meet job demands
	4. Client has fatigue management strategies
	5. Client has coping skills/strategies to deal with workplace interpersonal, organizational and task demands
	6. Client has confidence in ability to return to work
**Environmental outcome**	**Performance objectives**
**RTW plan is in place**	1. GRTW schedule is designed and agreed upon by stakeholders
	2. Workplace accommodations are provided
	3. Client’s strengths and limitations are assessed for job match

GRTW = gradual return-to-work.

Tolerance to work hours (performance objective 2) is a common objective to both forms of work hardening but is achieved in different ways through related performance objectives 3 and 4. For example, pain is often a limiting factor for an injured worker’s work tolerance whereas (mental) fatigue often limits work tolerance for persons with depression.

Attention now shifts to focus solely on the CWH intervention under study. The IM structure is used to further deconstruct CWH in order to enhance the understanding of the intervention and to provide a systematic way of describing the intervention and the underlying theory.

#### Tasks 3 and 4: Select determinants and Create matrices of change objectives

By breaking down each performance objective into its learning objectives (for the behavioural outcome) and change objectives (for the environmental outcome) important and changeable behaviour determinants are selected. This results in matrices of change objectives that are specific to each performance objective. Determinants selected in this current analysis are based on those set out by Bartholomew et al. [16] as well as those used by other researchers [17,22,41,46-48].

The determinants of *attitude*, *norms*, and *self-efficacy* can be traced to the de Vries ASE model of behaviour intention [49]. This model suggests that a person’s intention to perform a certain behaviour is determined by personal conceptions regarding the behaviour (attitude), the social pressures by others regarding the behaviour (norms), and personal belief in one’s ability to engage in the behaviour (self-efficacy). The realization of the behaviour is dependent on a positive intention but also requires the person having the skills/abilities to carry out the behaviour. This model is based on the Fishbein and Ajzen model for change behaviour [50] and Bandura’s Social Learning Model [51]. These three determinants, in addition to *risk perception and knowledge*, have been successfully utilized by other researchers applying IM to RTW interventions [22,41]. The determinant o*utcome expectations* has been studied by Bartholomew et al. [16,17] and is also included in the current analysis. *Skills* is also used as a determinant based on de Vries’ [49] contention that this is a related factor to behaviour change and the assertion by Kok et al. [46] that having the necessary skills to perform the behaviour is among the necessary and sufficient determinants for behaviour change.

The environmental determinants were selected based on evidence from the literature related to determinants already used in the development of RTW interventions for employees with musculoskeletal conditions as well as for employees with mental health problems [22,41] in addition to other related literature [17]. These include *norms*, *support*, *resources*, *organizational climate*, and *safety and equality*. In this paper, the concept ‘*norms’* is used to refer to the norms in the client’s workplace; most notably, related to the job tasks. S*afety and equality* refers to feeling secure in one’s workplace. It includes an environment which is psychologically ‘safe’ from provoking relapse where everyone is treated fairly and given what they need to succeed at their work (e.g., accommodations, if required) and an ‘equal’ environment is one where one’s human rights are considered. The accommodation process addresses both these aspects and is therefore an inherent part of this external determinant.

Table 4 presents an example of *learning objectives* that are associated with the performance objective: “*Client identifies RTW barriers, concurs with and commits to treatment plan*”. This performance objective is one among the six already presented that are associated with the behavioural outcome: *Client is ready to RTW*. Determinants of behaviour change are presented across the top of the matrix and include *risk perception and knowledge*, *attitude*, *skills*, *self-efficacy*, and *outcome expectations*. For each determinant, a change objective is created that links that determinant to the performance objective. The same process is applied with each of the other performance objectives resulting in a matrix being created by linking each determinant with each performance objective.

**Table 4:** **Example of learning objectives based on combination of performance objective and determinants**

**Performance objectives for client**	**Learning objectives**				
	**Risk perception and knowledge**	**Attitude**	**Skills**	**Self-efficacy**	**Outcome expectations**
Client identifies RTW barriers, concurs with and commits to treatment plan.	Client identifies occupational performance issues and how they impact RTW readiness.	Client understands nature of intervention and has a positive attitude towards it.	Client recognizes occupational performance issues.	Client believes occupational performance issues can be overcome.	RTW barriers are addressed.

Table 5 presents an example of *change objectives* which are associated with the performance objective: “*GRTW is designed and agreed upon by stakeholders*”. This performance objective is one among the three already presented that are associated with the environmental outcome: *RTW plan is in place*. Determinants of behaviour change are presented across the top of the matrix and include *norms*, *support*, *resources*, *organizational climate*, and *safety & equality*. For each determinant, a change objective is again created linking that determinant to the performance objective. The same process is applied with each of the other performance objectives resulting in a matrix being created by linking each determinant with each performance objective.

**Table 5:** **Example of change objectives based on combination of performance objective and determinants**

**Performance objectives for environment**	**Change objectives**				
	**Norms**	**Support**	**Resources**	**Organizational climate**	**Safety & Equality**
GRTW is designed and agreed upon by stakeholders.	OT designs a typical GRTW schedule (e.g., 6–8 weeks).	OT reviews GRTW with client and addresses questions/concerns.	Roles of OT, insurer, and employer in GRTW process are clearly delineated.	Insurer reviews GRTW with employer for clarity and buy-in.	RTW concerns are addressed; questions answered.
					GRTW plan is accepted by client and all stakeholders.

### Step 3: Theory-based intervention methods and practical applications

Step 3 of the IM process involves choosing methods and practice strategies that are theoretically grounded and clarifying the underlying mechanisms that contribute to change objectives and ultimately program objectives [17]. This aligns with what Bartholomew et al. [17] describe as “the causal chain from determinants to objectives to methods to applications” (p. 313) which they note is often not reported in program descriptions; hence, it is difficult to judge the theory and evidence base behind what planners have chosen to include in their intervention. This step involves studying each determinant and the methods of behaviour change related to each determinant both at the individual and the environment level. It is precisely this step, discussed below, that explores methods and theories and links them to the tools/materials used in the CWH intervention. In so doing, a better understanding of the theoretical underpinnings of the CWH process that can explain the intervention outcome – *RTW preparation for people with depression –* is achieved*.* This is consistent with the goals of process evaluation which espouses to study program implementation in order to understand the relationship between program elements and program outcome(s) [19].

The detailed analysis of IM Step 3 provides insight into how each determinant relates to a theoretical method, a strategy, and ultimately to CWH tools. Matching practical applications to theory has affirmed strategies such as collaboration, targeted questioning, engagement in meaningful activity, empowerment, and education. Tools and materials are understood as having been selected as concrete and tangible ways of implementing a particular strategy. These items ultimately comprise the elements of the CWH intervention (Table 6).

**Table 6:** **Example of determinants linked to theories and practical strategies**

**Determinant**	**Methods (from theory)**	**Theory**	**Strategy**	**Tools/Materials**
**(L) = Learning**				
**(C) = Change**				
**Risk perception & knowledge (L)**	Personalize risk	CMOP-E [52]	Client-centred	Guided questions
		PEO [53]	Collaboration	Brochures, handouts
			Targeted questioning	Discussion of occupational performance issues/barriers to RTW
			Occupational performance issues	
			Meaningful occupation/activity	
			Written & verbal information	
	Discussion	ELM [54,55]	Discussion	Personalized discussions
	Elaboration		Self-reflection	
**Self-efficacy (L)**	Enactive mastery experiences	Bandura’s Social Learning Theory [51]	Personal performance accomplishments of tasks	Work simulations/activity selection
	Verbal persuasion	Rotter’s Locus of Control [56]	Reinforcing messages regarding capabilities	Task analysis
				Graded activity
	Engagement in meaningful activity	Recovery Model [57,58]	Empowerment	Exploration of linkages between performance, self-efficacy, occupational performance
		CMOP-E		
				Discussions – strengths, work ability
		PEO		
	Self-monitoring of behavior	Theory of Self-Regulation [59]	Monitoring of newly acquired skills (e.g., assertiveness)	Client keeps a record of situations in which they practiced assertiveness to review with OT
**Norms (C)**	Cognitive skill development	The Dynamic Interactional Model of Cognition [60,61]	Approach to task	Job description
			Development of task skill to match task demands	Functional assessments
				Task analysis
				Work simulations
				Graded activity
	Participation in meaningful activity	CMOP-E	Adoption of work routine	GRTW schedule
		PEO		
	Intrapersonal skill development	Appraisal Model of Coping [62]	Assertiveness training	Role plays
			Stress management	Vignettes
		Bandura’s Social Learning Theory	Education	Audiovisual resources
		Rotter’s Locus of Control	Repetition	
			Practice	
			Problem-solving	
**Safety & equality (C)**	Duty to accommodate process	CMOP-E	Task analysis	Job descriptions
		CMCE	Functional analysis	Work simulations
		CPPF	Job demands analysis	Accommodation process
				Discussions with client, insurer, employer

The detailed work of IM Step 3 establishes the relationship among program tools/materials, determinants, and performance objectives. Table 7 illustrates how select CWH tools/materials are linked to and shown to support a determinant and, in turn, how the determinant is linked to and is shown to support a performance objective. This draws a much clearer association between performance objectives and the program elements (i.e. tools/materials) which together contribute to the overall CWH program objective of RTW.

**Table 7:** **Example of CWH tools mapped to performance objectives**

**Tool**	**Determinant**	**Learning objective**	**Performance objective(PO)**
**Work simulations**	Self-efficacy	Client experiences markers of improved work performance contributing to the belief that s/he is ready to RTW	Client has the confidence in his/her ability to RTW
			(PO #6)
**Videos, role plays, coaching**	Skills	Client has the ability to apply assertiveness skills to personal & work situations	Client has the coping skills to deal with workplace stress (PO #5)
**Education**	Risk perception & knowledge	Client learns the value of pacing	Client has fatigue management strategies
			(PO #4)

Special attention is drawn in this analysis to ‘s*elf-efficacy’* as a determinant of behaviour change because it is linked to the tool ‘work simulations’ which are a fundamental element of CWH. Work simulations are used to simulate a client’s pre-disability task demands in order to facilitate cognitive skill development. They are developed by the OT, with input from the client, through a task analysis of the client’s work duties and are graded in complexity as the client progresses in the CWH process. Engagement in meaningful occupation is the central process of change in CWH and guides the occupational therapy process [1,2]. Indeed ‘engagement’ is a hallmark of the occupational therapy profession and a key concept of the Canadian Model of Occupational Performance and Engagement (CMOP-E) which presents an occupational perspective that includes and extends beyond occupational performance to include engagement [52]. In this way, the link is made between intervention tools and determinants through underlying occupational therapy theory which guides practice through methods and strategies.

The determinant ‘*self-efficacy*’ is also informed by Bandura’s Social Learning Theory [51] which indicates the importance of a person’s personal mastery expectations regarding a desired behaviour. Self-efficacy refers to one’s beliefs about one’s ability to perform a specific behaviour. Individuals with low self-efficacy will likely avoid situations and/or not engage in behaviours that they feel unable to cope with or perform. Individuals with high levels of self-efficacy will be more likely to engage in behaviour in which they feel more confident to perform and will likely persist with behaviours that may become difficult which in turn increases their self-efficacy expectations further. Through engaging in meaningful work simulations in CWH, an individual gains mastery through personal performance accomplishments which instills confidence and reinforces one’s sense of self-efficacy. Learning through experience is one of four main sources of self-efficacy that Bandura highlighted in his theory and has been noted to be the most effective [51,63].

Drawing from the work of van Oostrom et al. [22] and Vermeulen et al. [41], the RTW process requires not only one’s attitude, social influence, and self-efficacy to drive one’s intention for behaviour change but must also consider the influence of barriers and facilitators together with knowledge and skills to achieve RTW. With this in mind, focus on occupational performance issues through targeted questioning and discussions contribute to the role that ‘*knowledge’* plays as a determinant of RTW in the CWH process. Clients are engaged in RTW preparation by examining their occupational performance issues as they relate to their cognitive functioning and skills, their environment (home/work), and the actual job demands at the workplace. This is consistent with client-centred models which stress that occupational performance and engagement result from the dynamic relationship between people, their occupations, and the environments in which they live, work, and play [52,53].

### Step 4: Intervention program and Step 5: Adoption and implementation

IM Step 4 and Step 5 involve program creation and implementation and are not applicable in this analysis where IM is being used to deconstruct an existing intervention.

### Step 6: Evaluation

IM Step 6 can be applied as it would be in the usual IM process for a newly designed program for the purpose of program monitoring and evaluation. This is indeed an important step that guides and enriches health education research through potential program enhancement [16,17].

Using IM to deconstruct CWH provides increased insight into the intervention’s underlying models and theory of change which, according to Bartholomew et al. [17], drives the evaluation process. In addition, the current analysis highlights the relationship between intervention change/learning objectives, behaviour change techniques, and intervention tools/materials within the over arching theoretical base of the intervention. This allows for evaluation of process and effect of the intervention to determine if any changes are needed for program improvement [43,64].

Through this analysis, self-efficacy (and its impact on one’s belief in one’s work ability) as well as fatigue emerged as critical RTW success factors; however, it became apparent that no standardized measurement tools were implemented to measure either of these constructs. The inclusion of such measurement tools emerged as an intervention improvement to gage work ability and fatigue status from intake (baseline) to discharge (program completion). Scores on standardized measures and difference scores may indeed have implications for RTW success. This is being studied in the first author’s current research study.

Although a focused process evaluation is not within the scope of this analysis, a few aspects of the process evaluation approach are worth noting. Implementation fidelity refers to the fidelity with which a program is implemented and has been discussed as an important factor mediating between interventions and their outcomes [19,20]. This may indeed influence the success of the CWH intervention and would need to be addressed in knowledge transfer activities related to the CWH intervention (e.g., workshops, training manuals). Contextual issues [19] include resource considerations such as adequately trained therapists to implement the CWH intervention, stakeholders that would be willing to support CWH for their clients/employees, and the physical environment consistent with the ideals of CWH (e.g., community-based, non-clinical).

Deconstruction of an existing intervention contributes to the field of health research. Feedback from this analysis builds on the fundamental goal of evaluation -- program enhancement -- by adding a layer of insight into the intervention’s underlying theoretical models and strategies which can then strengthen the body of knowledge used to address other health issues. Indeed, learnings obtained regarding models and in vivo strategies have the potential to influence their usage and possible further evolvement.

## Discussion

This paper provided a theoretical analysis of CWH by adapting the IM process to deconstruct the treatment intervention. CWH is a treatment intervention designed to prepare people to return to work following a depression and is based on established work hardening principles. Providing opportunity for mastery prior to returning to work helps to mitigate the prevalence of stigma against depression in today’s workplaces [65].

As part of a strategy to familiarize the health care and vocational rehabilitation community with this tailored approach, communication, knowledge development, research and evaluation of the intervention is being undertaken and is enhanced by the systematic development of a formal program description. IM provided the framework with which to methodically analyze CWH to gain a better understanding and appreciation of its component parts and underlying theoretical foundation. At the same time, CWH served as an illustrative example of the adapted IM process which has been formulated and presented here.

The structured IM process provided a roadmap for the detailed intervention analysis of CWH that included establishing performance objectives and change objectives. Matrices were developed that linked determinants of behaviour change to performance objectives resulting in change objectives that were then mapped to CWH tools that are the basic elements of the intervention. Application of the IM protocol enabled the linking of strategies and tools to theories to analyze the intervention’s underlying mechanisms that are believed to impact the desired intervention outcomes. Empirical study of the efficacy of the intervention and client feedback on useful intervention elements is currently underway, and findings should provide added insight and understanding into this intervention.

The current analysis contributes to the scientific literature and the clinical field by providing insight into CWH that is not only descriptive but is also theory-based thereby enhancing understanding of this treatment intervention. The sequential deconstruction of CWH has the potential to highlight gaps in the intervention which can then be addressed to improve the intervention and treatment outcome. This is consistent with the feedback loop that is inherent in the structured IM framework which consists of program evaluation for continued intervention improvement.

The IM structure provides a common language to share intervention content and rationale [64] and, indeed, using the IM tool to deconstruct an existing intervention contributes to the field of research through this shared language. In this way, the scientific knowledge base is enhanced through communication of an intervention’s underlying theoretical models, strategies, and program tools which can then be applied to other treatment interventions and health concerns thereby benefiting the broader research and clinical communities. In addition, intervention analysis encompasses in-depth study of intervention models and practical strategies that can lead to improvement and evolvement of these elements which can also enhance the body of scientific and clinical knowledge.

Although IM was originally designed as a program development tool, by embracing the application of its protocol as described in this paper, other existing interventions can be deconstructed to gain insight into their programs for the purpose of program description, evaluation and ultimately improved health outcomes. Adoption by more program designers and researchers results in a larger pool of interventions being systematically studied with findings informing the health research community and facilitating the exchange of best practices.

## Conclusions

The use of IM to deconstruct an existing intervention proved to be a useful tool to systematically analyze and describe the intervention’s theoretical underpinnings which promotes knowledge sharing and lays the foundation for intervention evaluation and improvement. This approach has implications for other existing interventions where using a common systematic protocol provides a shared language enhancing knowledge exchange among practitioners and the research community.

## Abbreviations

CWH: Cognitive work hardening
IM: Intervention mapping
RTW: Return to work
PWH: Physical work hardening
OT: Occupational therapist
GRTW: Gradual return-to-work
CMOP-E: Canadian Model of Occupational Performance and Engagement
PEO: Person Environment Occupation
ELM: Elaboration Likelihood Model
CMCE: Canadian Model of Client-Centred Enablement
CPPF: Canadian Practice Process Framework
